# The association between labor epidural analgesia and postpartum depression: a systematic review and meta-analysis

**DOI:** 10.1186/s12905-020-00948-0

**Published:** 2020-05-11

**Authors:** Marcela Almeida, Katherine A. Kosman, Mark C. Kendall, Gildasio S. De Oliveira

**Affiliations:** 1 Department of Psychiatry, Brigham and Women’s Hospital, Harvard Medical School, Boston, MA USA; 2 Department of Anesthesiology, The Warren Alpert Medical School of Brown University, Providence, RI USA

**Keywords:** Labor analgesia, Postpartum depression, Maternal mental health, Systematic review

## Abstract

**Background:**

Previous studies have demonstrated that appropriate treatment for postoperative pain can lead to improvement in depressive symptoms, however the association between adequate intrapartum pain control and the development of postpartum depression is not clear. The purpose of the study was to examine the effects of labor epidural analgesia and postpartum depression.

**Methods:**

We performed a quantitative systematic review in compliance with the PRISMA statement. We conducted a search of PubMed, Embase, the Cochrane Database of Systematic Reviews and Google Scholar databases. The primary outcome was a positive screen of postpartum depression among women who received labor epidural analgesia up to 3 months into the postpartum period. Meta-analysis was performed using the random effect model.

**Results:**

Of the 148 studies available, 9 studies with 4442 patients were included in the analysis. The use of labor analgesia on positive depression screen compared to control revealed no significant effect, OR (95% CI) of 1.02 (0.62 to 1.66, *P* = 0.94).

**Conclusion:**

Based on current literature, the use of epidural analgesia for pain relief during labor doesn’t appear to affect the likelihood of postpartum depression. Future studies are warranted to further investigate these findings and identity other possible preventative interventions that reduce postpartum depression.

## Background

Postpartum depression is one of the most common complications of childbirth, affecting one in seven women [1]. Untreated postpartum depression (PPD) is associated with an array of negative outcomes including suicide, infanticide and long- term behavioral consequences to the newborn (e.g., depression, violence and aggression) [2, 3]. While several risk factors that contribute to PPD have been identified (e.g., psychosocial stressors, family and spousal support, income, and marital), very few risk factors are modifiable [4, 5].

Pain is a well-known risk factor for the development of depression [6]. Nonetheless, the inclusion of peripartum pain as a potential risk factor for the development of postpartum depression has only recently been identified and more extensively studied [7]. Several studies have evaluated the association between the use of labor epidurals and postpartum depression leading to conflicting results. It is currently unknown if optimal peripartum pain control using epidural analgesia is associated with a reduction in the incidence of postpartum depression.

Given the strong association between pain and depression, one can argue that optimal labor analgesia (e.g. use of epidural infusions of local anesthetics and opioids) may reduce the incidence of postpartum depression.

The main objective of the current study is to evaluate a possible association between optimal labor analgesia using epidurals and the development of postpartum depression. We hypothesized that patients who received epidural analgesia would develop a lower incidence of postpartum depression.

## Methods

### This systematic review was conducted following the guidelines of the preferred

Reporting Items for Systematic Reviews (PRISMA) statement [8]. Institutional review board approval and patient consent were not required.

#### Systematic search

Published articles evaluating the effects of peripartum pain control via labor analgesia on postpartum depression were searched using the electronic PubMed database, Embase, the Cochrane Database of Systematic Reviews and Google Scholar up to July 2019 (Additional file 1). Key words and MeSH descriptor terms “pain”, “labor”, “analgesia” and “depression”’ were used individually and in various combinations. An attempt to identify additional studies was made by reviewing the bibliographies from identified studies. No language restrictions were used. Unpublished studies were not investigated. There was no limitation on sample size.

#### Inclusion and exclusion criteria

The inclusion and exclusion criteria were defined prior to the initiation of the systematic review. We included peer-reviewed observational- prospective trials that investigated peripartum pain control via labor analgesia in women who were experiencing postpartum depression up to 12 months postpartum as defined by the Diagnostic Statistical Manual of Psychiatric disorders (DSM) [9]. We excluded studies in which woman experienced early pregnancy loss or stillbirth. Studies that did not implement the use of formal screening measure for depression or did not report specific postpartum depression assessments were omitted.

#### Selection of the included clinical studies and data extraction

The investigators individually assessed the abstracts and outcomes extracted from the initial query. Articles that did not meet the inclusion criteria or fulfilled the exclusion criteria were omitted. Discrepancies among the reviewers were finalized by discussion and if resolution was not met, the final decision was determined by an additional investigator who was not present or aware of the discussions held by the previous investigators.

The authors independently evaluated the full manuscripts of all potentially eligible trials and performed data extraction using a data collection form specifically developed for this review. Data explored in the literature included the study design, number of participants, inclusion and exclusion criteria for the study, sample size, number of subjects in treatment groups, type of labor analgesia administered, dose of analgesia administered, psychiatric screening tools (Edinburgh Postnatal Depression Scale, DSM diagnosis) time period of postpartum depression assessments, and the conclusion to each study. Disagreements on extracted variables were resolved by consulting with an additional investigator.

#### Methodological quality assessment of clinical studies

The investigators assessed the quality of the included studies using the Newcastle-Ottawa Quality Assessment Scale [10]. This tool assesses aspects regarding population/sampling methods, exposure/outcome collections, as well as statistical matching/adjustments of the data. Quality scores were assigned for each domain, and an overall score was assigned for each study, with a maximum possible score of 9. Studies with a score of ≥7 were categorized as high-quality studies, and those with a score of < 7 were categorized as low-quality studies.

#### Primary outcome

A positive screen of postpartum depression among women who received or did not receive labor epidural analgesia up to 3 months into the postpartum period.

#### Meta-analyses

For positive postpartum depression screen (dichotomous data), the Peto odds ratio (to account for the potential of zero counts in the cells for low frequency outcomes) and 99% CI are reported. A significant effect compared to control required that the 99% CI for continuous data did not include zero and for dichotomous data, the confidence interval did not include 1.0. Due to the inclusion of different study populations, we choose to use the random effects model to generalize our findings to studies not included in our meta-analysis. The random effect model has an advantage to the fixed effect model since it does not rely on the assumption that a true effect size is the same in all combined studies [11]. Publication bias was evaluated by examining for asymmetric funnel plot using the Egger’s regression test [12, 13]. A one-sided *P* < 0.05 was considered as an indication of an asymmetric funnel plot.

Of the included studies, heterogeneity was considered to be high if the I^2^ statistic was greater than 50%. If heterogeneity was high, we performed a sensitivity analysis by removing individual studies according to its methodological quality and examining its effect on the overall heterogeneity. A *P* value < 0.05 was required to reject the null hypothesis. Analyses was performed using Stata version 15 (College Station, Texas) and Comprehensive Meta-analysis software version 3 (Biostat, Englewood, NJ).

## Results

**Fig. 1:**
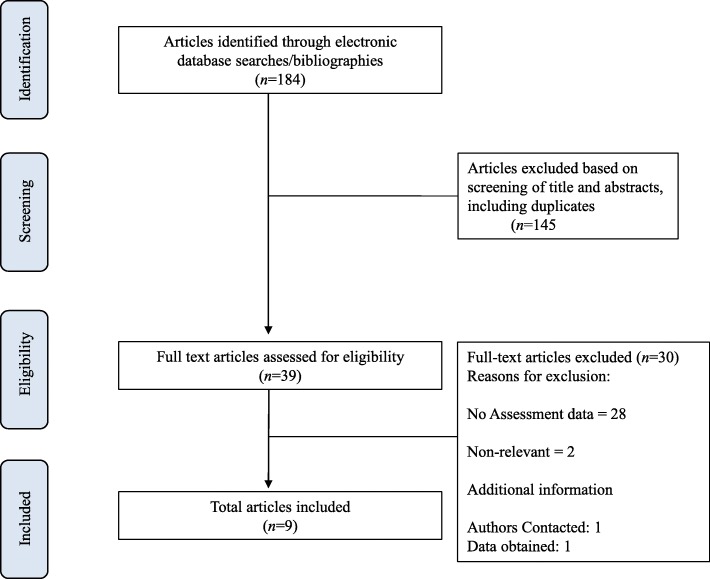
Flow chart of the selection of studies

The initial search yielded 184 studies and 145 articles were omitted that did not fulfill the inclusion criteria upon further evaluation of the study abstracts. The full text of 39 articles were evaluated, and 30 articles were excluded because they did not meet the inclusion criteria. The flowchart of selected studies with specific reasons for exclusion is shown in Fig. 1.

A total of 9 studies that were published between 2014 and 2019 with 4442 women were eligible and included in the analysis (Table 1) [14–22]. The overall incidence of a positive screen for postpartum depression in the total sample was 9.51%. All nine studies reported on postpartum depression within 3 months following delivery. All studies used the Edinburgh Postnatal Depression Scale (EPDS) as the screening tool for presence of postpartum depression. The methodological quality of included trials is presented in Table 2.

**Table 1:** Summary of study characteristics included in analysis

Author	Year	Design	Intervention/Control	Recruitment	PPD Time Period	Measure of postpartum depression	Results
Ding et al. [14] Origin: China	2014	Observational; prospective cohort	107/107	Patient decided to have ELA or no pain relief at all	6 w	Edinburgh Postnatal Depression Scale (cutoff score ≥ 10)	PPD was significantly less in LEA group (*P* < 0.001)
Eckerdal et al. [15] Origin: Sweden	2019	Longitudinal cohort study	800/703	Patients recruited prior to delivery	6 w	Edinburgh Postnatal Depression Scale (cutoff score ≥ 12)	No difference between LEA and PPD
Gaillard et al. [16] Origin: France	2014	Observational	217/47	Patients recruited prior to delivery	8 w	Edinburgh Postnatal Depression Scale (cutoff score ≥ 12) Diagnostic Interview for Genetic Studies	No difference between LEA and PPD
Nahirney et al. [17] Origin: Canada	2017	Observational; prospective cohort	88/107	Patients recruited on post-delivery	6 w	Edinburgh Postnatal Depression Scale (cutoff score ≥ 10)	No difference between LEA and PPD
Orbach-Zinger et al. [18] Origin: Israel	2018	Observational; prospective	604/394	Patients recruited on post-delivery day 1	6 w	Edinburgh Postnatal Depression Scale (cutoff score ≥ 10)	No significant increase in PPD among LEA
Riazanova et al. [19] Origin: Russia	2018	Observational	107/103	Patient decided to have ELA or no pain relief at all	6 w	Edinburgh Postnatal Depression Scale (cutoff score ≥ 10)	No significant increase in PPD among LEA
Suhitharan et al. [20] Origin: USA	2016	Observational; case-control	329/150	Patients recruited in postnatal period (LEA or entonox/pethidine)	8 w	Edinburgh Postnatal Depression Scale (cutoff score ≥ 10) + DSM-IV Criteria Interview via psychiatrist	PPD was significantly less in LEA group (*P* < 0.008)
Tobin et al. [21] Origin: USA	2016	Observational; prospective secondary analysis	50/15	Medical records reviewed for LEA/no LEA	8 w	Edinburgh Postnatal Depression Scale (cutoff score ≥ 10)	Epidural analgesia did not reduce occurrence of PPD.
Zhang et al. [22] Origin: China	2018	Observational	213/301	Patients decided on 3 groups of pain relief; doula, transcutaneous electrical nerve stimulation, or epidural analgesia.	4 w	Edinburgh Postnatal Depression Scale (cutoff score ≥ 10)	Epidural analgesia did not reduce occurrence of PPD.

**Table 2:** New-castle Ottawa methodological quality of included non-randomized studies

**Cohort Studies**	**Selection**				**Comparability**	**Outcome**			**Score**
	**Representative-ness of exposed cohort**	**Selection of the non-exposed cohort**	**Ascertainment of exposure**	**Outcome of interest was not present at start of study**	**Comparability based on design or analysis**	**Assessment of outcome**	**Adequacy of follow up of cohorts**	**Loss to follow up < 20%**	
Ding 2014 [14]	1	1	1	0	2	0	1	1	7
Eckerdal 2019 [15]	1	1	1	0	2	0	1	1	7
Gaillard 2014 [16]	1	1	1	0	2	1	1	1	8
Nahirney 2017 [17]	1	1	1	1	2	0	1	1	8
Orbach-Zinger 2018 [18]	1	1	1	1	2	1	1	1	9
Riazanova 2018 [19]	1	1	1	0	0	1	0	0	4
Tobin 2016 [21]	1	1	1	0	0	0	1	0	4
Zhang 2018 [22]	1	1	1	1	2	1	0	1	8
**Case-Control Study**	**Selection**				**Comparability**	**Outcome**			**Score**
	**Adequate definition of cases**	**Representativeness of cases**	**Selection of controls**	**Definition of controls**	**Comparability based on design or analysis**	**Ascertainment of exposure**	**Same method of ascertainment for cases and controls**	**Same response rate for both groups**	
Suhitharan 2016 [20]	1	1	1	1	2	1	1	1	9

### Methodological quality of studies

The quality of studies ranged from 4 to 9 points, with a median of 8 (7 to 8). There were7 studies that scored ≥7 representing high-quality with 2 studies scoring below 7 representing low quality studies. Five studies did not clearly demonstrate the presence of depression prior study enrollment. Two studies scored a zero for comparability on basis of analysis for not adjusting for known confounding variables such as past or family history of depression or an additional factor including difficulty during childbirth. The assessment of outcome was self-reported in four studies. In two studies, the adequacy of study follow-up was not clearly reported.

### Postpartum depression

**Fig. 2:**
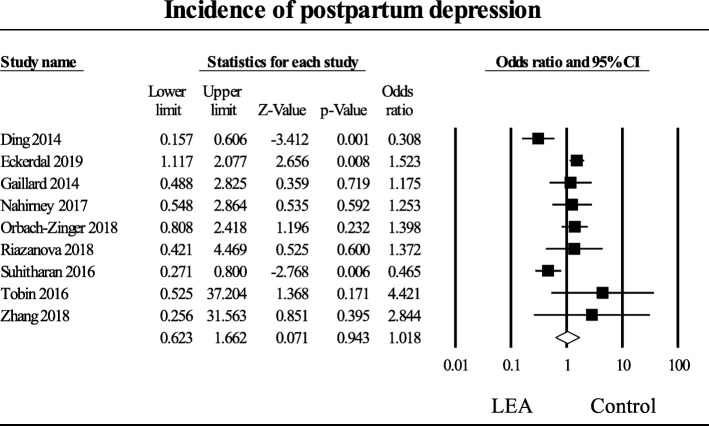
Incidence of postpartum depression. Random-effects meta-analysis evaluating the effect of labor epidural analgesia on postpartum depression compared to control. Squares to the right of the middle vertical line indicates that labor epidural analgesia was associated with increased odds of postpartum depression, whereas squares to the left of the middle vertical line show that labor epidural analgesia was associated with decreased odds of postpartum depression. The horizontal lines represent the 95% CI and the diamond shape represents the overall effect of labor epidural analgesia on postpartum depression compared to control. CI = confidence interval

In the nine studies that reported on postpartum depression [14–22], the aggregated effect of the studies that investigated postpartum depression among women who received labor epidural analgesia compared to control did not reveal a significant effect, OR (95% CI) of 1.02 (0.62 to 1.66) (*P* = 0.94), (Fig. 2).

Heterogeneity was high, I^2^ = 74%. An examination of the funnel plot did not reveal asymmetry; Eggers regression test revealed a one-sided *P* = 0.46. A sensitivity analysis removing the low methodological quality studies (score < 7) did not reduce the heterogeneity.

## Discussion

The most important finding of our investigation was the lack of association between the use of epidural analgesia and postpartum depression. Patients who received epidural analgesia were not less likely to develop postpartum depression compared to patients who did not received epidural analgesia. Although individual studies have demonstrated a significant association between epidural analgesia and postpartum depression, the aggregated association was not significant despite a large sample size of over 4400 patients.

Our results are clinically important since optimal labor analgesia has been recently proposed as potential modifiable risk factor for postpartum depression by multiple investigators [23, 24]. This hypothesis has been based on the strong association between chronic pain conditions and the development of mood disorders [14, 25, 26]. It is possible that the acute nature of labor pain is not enough to lead to the development of postpartum depression.

Epidural infusions are considered the gold standard for labor analgesia and the current use of ultrasonography and other modalities to confirm epidural catheter placement has become popular among clinicians [27–29]. Moreover, the use of epidural infusions has been consistently demonstrated to improve the mother’s experience through labor [30, 31]. Nonetheless, epidural analgesia is often not continued much longer beyond child delivery and it has yet to be investigated if the use of epidural analgesia can reduce postpartum chronic pain.

Although we did not detect an association between postpartum depression and the use of labor epidural analgesia, we cannot exclude a potential benefit of epidural analgesia on the severity of mood oscillations after labor. Labor and postpartum pain can affect the ability of mothers to bond with their infants via attachment and caregiving during that critical developmental period. It is possible that labor analgesia can reduce the incidence of postpartum blues, and this may lead to a benefit to parturient. Future studies examining labor pain and postpartum depression should include not only a dichotomous outcome, but also the specific depression score.

While the question on whether labor analgesia may reduce the likelihood of postpartum depression remains unanswered, the importance of universal maternal mental and physical health screening is well established. Women should be routinely screened for intra-labor and postpartum pain and receive proper pain management, and clinicians should be aware of the impact of pain in the development of postpartum depression. Given that many risk factors for postpartum depression cannot be modified, it is worth elucidating whether pain is one of the modifiable ones so that the risks associated with intrapartum analgesia can be better weighed against its benefits.

Our study should only be interpreted within the context of its limitations. First, no universally accepted time point for PPD screening and this may have resulted in variations on the detection of postpartum depression among the studies. Second, although epidural analgesia is the gold standard to minimize labor pain, it is possible that patients have received other less effective techniques that minimized the potential effect of an epidural. Last, observational studies cannot account for unknown confounders that can only be adequately managed by randomized clinical trials. Nonetheless, it is unlikely that a future study will be able to randomize patients to epidural or non-epidural techniques given the ethical implications.

## Conclusions

In summary, we did not detect an association between epidural analgesia and the development of postpartum depression. Clinicians should not advocate for the use of an epidural as a potential strategy to reduce postpartum depression to patients. In addition, clinicians should utilize proven preventive strategies to minimize the development of postpartum depression on the mother and the newborn child.

## Supplementary information

**Additional file 1.**

## Data Availability

Not applicable.

## Abbreviations

CI: Confidence interval
DSM: Diagnostic statistical manual of psychiatric disorders
EPDS: Edinburgh postnatal depression scale
LEA: Labor epidural analgesia
MeSH: Medical subject headings
OR: Odds ratio
PPD: Postpartum depression
PRISMA: Preferred reporting items for systematic reviews assessment

## References

[1] Wisner KL, Sit DKY, McShea MC (2013). Onset timing, thoughts of self-harm, and diagnoses in postpartum women with screen-positive depression findings. JAMA Psychiatry.

[2] O'Higgins M, Roberts IS, Glover V, Taylor A (2013). Mother-child bonding at 1 year; associations with symptoms of postnatal depression and bonding in the first few weeks. Arch Womens Ment Health..

[3] Dubber S, Reck C, Müller M, Gawlik S (2015). Postpartum bonding: the role of perinatal depression, anxiety and maternal-fetal bonding during pregnancy. Arch Womens Ment Health..

[4] Grace SL, Evindar A, Stewart DE (2003). The effect of postpartum depression on child cognitive development and behavior: a review and critical analysis of the literature. Arch Womens Ment Health..

[5] Lane WE, Cobert J, Horres CR, Strouch Z, Mehdiratta J (2019). Undetected uterine rupture during induction of labor for intrauterine fetal demise using epidural anesthesia. J Clin Anesth.

[6] Spinelli MG (2004). Maternal infanticide associated with mental illness: prevention and the promise of saved lives. Am J Psychiatry.

[7] Howell EA, Mora PA, Di Bonaventura MD, Leventhal H (2009). Modifiable factors associated with changes in postpartum depressive symptoms. Arch Womens Ment Health.

[8] Liberati A, Altman DG, Tetzlaff J (2009). The PRISMA statement for reporting systematic reviews and meta-analyses of studies that evaluate health care interventions: explanation and elaboration. J Clin Epidemiol.

[9] Association AP (1994). Diagnostic and statistical manual of mental disorders.

[10] Stang A (2010). Critical evaluation of the Newcastle-Ottawa scale for the assessment of the quality of nonrandomized studies in meta-analyses. Eur J Epidemiol.

[11] DerSimonian R, Laird N (1986). Meta-analysis in clinical trials. Control Clin Trials.

[12] Egger M, Smith GD, Schneider M, Minder C (1997). Bias in meta-analysis detected by a simple, graphical test. BMJ..

[13] Lovett-Carter D, Kendall MC, McCormick ZL, Suh EI, Cohen AD, De Oliveira GS. Pectoral nerve blocks and postoperative pain outcomes after mastectomy: a meta-analysis of randomized controlled trials. Reg Anesth Pain Med. 2019;9. 10.1136/rapm-2019-100658.. 10.1136/rapm-2019-100658 31401620

[14] Ding T, Wang D-X, Qu Y (2014). Epidural labor analgesia is associated with a decreased risk of postpartum depression: a prospective cohort study. Anesth Analg.

[15] Eckerdal P, Kollia N, Karlsson L (2020). Epidural analgesia during childbirth and postpartum depressive symptoms: a population-based longitudinal cohort study. Anesth Analg.

[16] Gaillard A, Le Strat Y, Mandelbrot L, Keïta H, Dubertret C (2014). Predictors of postpartum depression: prospective study of 264 women followed during pregnancy and postpartum. Psychiatry Res.

[17] Nahirney M, Metcalfe A, Chaput KH (2017). Administration of epidural labor analgesia is not associated with a decreased risk of postpartum depression in an urban Canadian population of mothers: a secondary analysis of prospective cohort data. Local Reg Anesth.

[18] Orbach-Zinger S, Landau R, Harousch AB (2018). The relationship between Women’s intention to request a labor epidural analgesia, actually delivering with labor epidural analgesia, and postpartum depression at 6 weeks: a prospective observational study. Obstetric Anesthesiol Anesth Analg.

[19] Riazanova OV, Alexandrovich YS, Ioscovich AM (2018). The relationship between labor pain management, cortisol level and risk of postpartum depression development: a prospective nonrandomized observational monocentric trial. Rom J Anaesth Intensive Care.

[20] Suhitharan T, Pham TP, Chen H (2016). Investigating analgesic and psychological factors associated with risk of postpartum depression development: a case-control study. Neuropsychiatr Dis Treat.

[21] Tobin CD, Wilson SH, Hebbar L (2016). Labor epidural analgesia and postpartum depression. Arch Depress Anxiety.

[22] Zhang Y, Johnston L, Ma D (2018). An exploratory study on the effect of labor pain management on postpartum depression among Chinese women. Ginekol Pol.

[23] Wisner KL, Stika CS, Clark CT (2014). Double duty: does epidural labor analgesia reduce both pain and postpartum depression?. Anesth Analg.

[24] Eisenach JC, Pan PH, Smiley R, Lavand’homme P, Landau R, Houle TT (2008). Severity of acute pain after childbirth, but not type of delivery, predicts persistent pain and postpartum depression. Pain..

[25] Lim G, Chelly JE (2015). More research is required to demonstrate a relationship between Intrapartum pain management and postpartum mood disorders. Anesth Analg.

[26] Pettersson FD, Hellgren C, Nyberg F, Åkerud H, Sundström-Poromaa I (2016). Depressed mood, anxiety, and the use of labor analgesia. Arch Womens Ment Health..

[27] Carvalho B, Seligman KM, Weiniger CF (2019). The comparative accuracy of a handheld and console ultrasound device for neuraxial depth and landmark assessment. Int J Obstet Anesth.

[28] Vernon TJ, Vogel TM, Dalby PL, Mandell G, Lim G (2019). Ultrasound-assisted epidural labor analgesia for landmark identification in morbidly obese pregnant women: a preliminary investigation. J Clin Anesth.

[29] Uchino T, Miura M, Matsumoto S, Shingu C, Kitano T (2018). Use of epidurography and computed tomography to identify misplacement of a section of an epidural catheter in the subarachnoid space. J Clin Anesth.

[30] Howell CJ, Chalmers I (1992). A review of prospectively controlled comparisons of epidural with non-epidural forms of pain relief during labour. Int J Obstet Anesth.

[31] Anim-Somuah M, Smyth R, Howell C (2005). Epidural versus non-epidural or no analgesia in labour. Cochrane Database Syst Rev.

