# Supplementary material for: The association between labor epidural analgesia and postpartum depression: a systematic review and meta-analysis
Source: BMC Womens Health. 2020 May 11;20:99. doi: 10.1186/s12905-020-00948-0 (PMC7216422; doi:10.1186/s12905-020-00948-0)
Supplement: Supplementary file 1 — Additional file 1. [file 12905_2020_948_MOESM1_ESM.docx]

**Appendix A**

Search Strategy

*PubMed*

(("labour"[All Fields] OR "work"[MeSH Terms] OR "work"[All Fields] OR "labor"[All Fields] OR "labor, obstetric"[MeSH Terms] OR ("labor"[All Fields] AND "obstetric"[All Fields]) OR "obstetric labor"[All Fields]) AND ("analgesia, epidural"[MeSH Terms] OR ("analgesia"[All Fields] AND "epidural"[All Fields]) OR "epidural analgesia"[All Fields] OR ("epidural"[All Fields] AND "analgesia"[All Fields]))) AND ("depressive disorder"[MeSH Terms] OR ("depressive"[All Fields] AND "disorder"[All Fields]) OR "depressive disorder"[All Fields] OR "depression"[All Fields] OR "depression"[MeSH Terms])

*Cochrane Database of Systematic Reviews*

labor epidural analgesia in Title Abstract Keyword AND depression in Title Abstract Keyword

*EMBASE*

('labor epidural analgesia' OR (('labor'/exp OR labor) AND epidural AND ('analgesia'/exp OR analgesia))) AND ('depression'/exp OR depression) AND [adult]/lim
